# Fertility and HIV following universal access to ART in Rwanda: a cross-sectional analysis of Demographic and Health Survey data

**DOI:** 10.1186/s12978-017-0301-x

**Published:** 2017-03-14

**Authors:** Eric Remera, Kimberly Boer, Stella M. Umuhoza, Bethany L. Hedt-Gauthier, Dana R. Thomson, Patrick Ndimubanzi, Eugenie Kayirangwa, Salomon Mutsinzi, Alice Bayingana, Placidie Mugwaneza, Jean Baptiste T. Koama

**Affiliations:** 1 Institute of HIV/AIDS Disease Prevention and Control, Rwanda Biomedical Center, Kigali, Rwanda; 2 Centers for Disease Control and Prevention, Kigali, Rwanda; 3 College of Medicine and Health Sciences, School of Public Health, University of Rwanda, Kigali, Rwanda; 4 Department of Global Health and Social Medicine, Harvard Medical School, Boston, USA; 5 Inshuti Mu Buzima/Partners in Health, Rwinkwavu, Rwanda; 6 National Institute of Statistics Rwanda, Kigali, Rwanda

**Keywords:** PMTCT, Africa, DHS, Evaluation, Women’s health

## Abstract

**Background:**

HIV infection is linked to decreased fertility and fertility desires in sub-Saharan Africa due to biological and social factors. We investigate the relationship between HIV infection and fertility or fertility desires in the context of universal access to antiretroviral therapy introduced in 2004 in Rwanda.

**Methods:**

We used data from 3532 and 4527 women aged 20–49 from the 2005 and 2010 Rwandan Demographic and Health Surveys (RDHS), respectively. The RDHSs included blood-tests for HIV, as well as detailed interviews about fertility, demographic and behavioral outcomes. In both years, multiple logistic regression was used to assess the association between HIV and fertility outcomes within three age categories (20–29, 30–39 and 40–49 years), controlling for confounders and compensating for the complex survey design.

**Results:**

In 2010, we did not find a difference in the odds of pregnancy in the last 5 years between HIV-seropositive and HIV-seronegative women after controlling for potential biological and social confounders. Controlling for the same confounders, we found that HIV-seropositive women under age 40 were less likely to desire more children compared to HIV-seronegative women (20–29 years adjusted odds ratio (AOR) = 0.31, 95% CI: 0.17, 0.58; 30–39 years AOR = 0.24, 95% CI: 0.14, 0.43), but no difference was found among women aged 40 or older. No associations between HIV and fertility or fertility desire were found in 2005.

**Conclusions:**

These findings suggest no difference in births or current pregnancy among HIV-seropositive and HIV-seronegative women. That in 2010 HIV-seropositive women in their earlier childbearing years desired fewer children than HIV-seronegative women could suggest more women with HIV survived; and stigma, fear of transmitting HIV, or realism about living with HIV and prematurely dying from HIV may affect their desire to have children. These findings emphasize the importance of delivering appropriate information about pregnancy and childbearing to HIV-infected women, enabling women living with HIV to make informed decisions about their reproductive life.

## Plain English summary

HIV infection is linked to decreased fertility and fertility desires in sub-Saharan Africa due to biological and social factors. We investigate the relationship between HIV infection and fertility or fertility desires in the context of universal access to antiretroviral therapy introduced in 2004 in Rwanda. In 2010, we did not find a difference in the odds of pregnancy between HIV-seropositive and HIV-seronegative women. We did, however, find that HIV-seropositive women under age 40 were less likely to desire more children compared to HIV-seronegative women. No associations between HIV and fertility or fertility desire were found in 2005. These findings suggest no difference in births or current pregnancy among HIV-seropositive and HIV-seronegative women, but that stigma, fear of transmitting HIV, or realism about living with HIV and prematurely dying may affect desire to have children among women under age 40. These findings emphasize the importance of delivering appropriate information about pregnancy and childbearing to HIV-infected women, enabling women living with HIV to make informed decisions about their reproductive life.

## Background

Infection with HIV has been linked to decreased fertility and fertility desires in sub-Saharan Africa due to both biological and social factors. Established HIV infection, typically occurring in older women, can compromise a woman’s ability to become pregnant or successfully carry a baby as a result of HIV-associated illnesses, fetal loss, reduced coital frequency, reduced spermatogenesis of the partner, and death of a partner [1, 2]. Furthermore, prior to wide availability of antiretroviral therapy (ART), clinicians were skeptical about encouraging HIV infected women to have children due to shortened life expectancy for the woman and high risk of HIV transmission to the child [3]. HIV infected women were also discouraged by families and communities from having children [4].

The advent of national HIV care and treatment programs that target the general HIV population and pregnant women may mediate the relationship between HIV infection and fertility. It is likely that ART removes biological barriers for an HIV infected woman to become a mother by reducing HIV associated illnesses [5, 6]. Importantly, the risk of HIV mother-to-child transmission (MTCT) has also been drastically decreased with national prevention of MTCT (PMTCT) programs [7]. Due to increased availability and knowledge of PMTCT programs which include family planning, there is wider support for HIV infected women to have children [4]. However, to date, there is little published research on fertility and fertility preferences of HIV infected women as compared to their uninfected counterparts in the context of expanded ART and PMTCT programs [1].

**Fig. 1:**
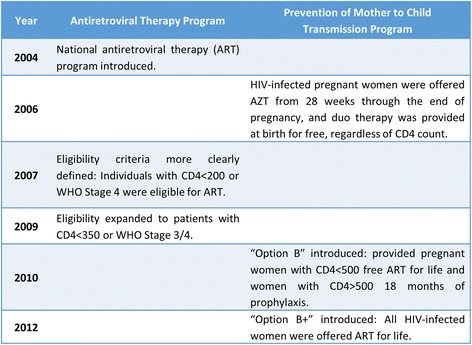
Evolution of the Rwanda HIV Antiretroviral Therapy and Prevention of Mother to Child Transmission Programs

In Rwanda, the national HIV care and treatment program was introduced in 2004 and policies for eligibility for ART and PMTCT have evolved over time (Fig. 1). The percentage of HIV seropositive women receiving ART during pregnancy to reduce the risk of MTCT in Rwanda has increased from 10% in 2004 to 64% in 2012 [8, 9]. Between 2001 and 2009, the mortality rate from HIV/AIDS in Rwanda was reduced by 50% or more across all age groups [Unpublished epidemiological population projections from UNAIDS Spectrum, 2014] and HIV incidence was reduced by more than 25% [9]. The Rwanda 2010 national guideline for clinical prevention of HIV/AIDS recommends voluntary HIV testing during antenatal care visits of pregnant women, and encourages testing and receipt of results together for couples. As a result, 98.2% of pregnant women were tested and received HIV results in 2010, and among them, 84% were tested and received results as a couple [10].

Major campaigns to get tested for HIV resulted in a more than threefold increase in the number of women who knew their status between 2005 and 2010 [10]. In 2010, an estimated 80% of eligible individuals were on ART and 83% of HIV infected pregnant women were receiving prophylaxis to reduce MTCT in PMTCT [10]. The goal of this paper is to examine the relationship between HIV infection and fertility in the context of expanded national ART and PMTCT programs in Rwanda.

## Methods

### Study population and design

Our primary analysis uses data from the 2010 RDHS [11] to assess the relationship between HIV and fertility among women in Rwanda. The 2010 RDHS is a nationally representative cross-sectional household survey of 13,671 women (15 to 49 years) and 6329 men (15 to 59 years) from 492 villages in Rwanda. The survey used a two-stage sampling design to produce separate estimates of key indicators for each of the 30 districts of Rwanda. All women aged 15 to 49 who were either permanent residents of the household or visitors in the household on the night before the survey were eligible to be interviewed.

**Fig. 2:**
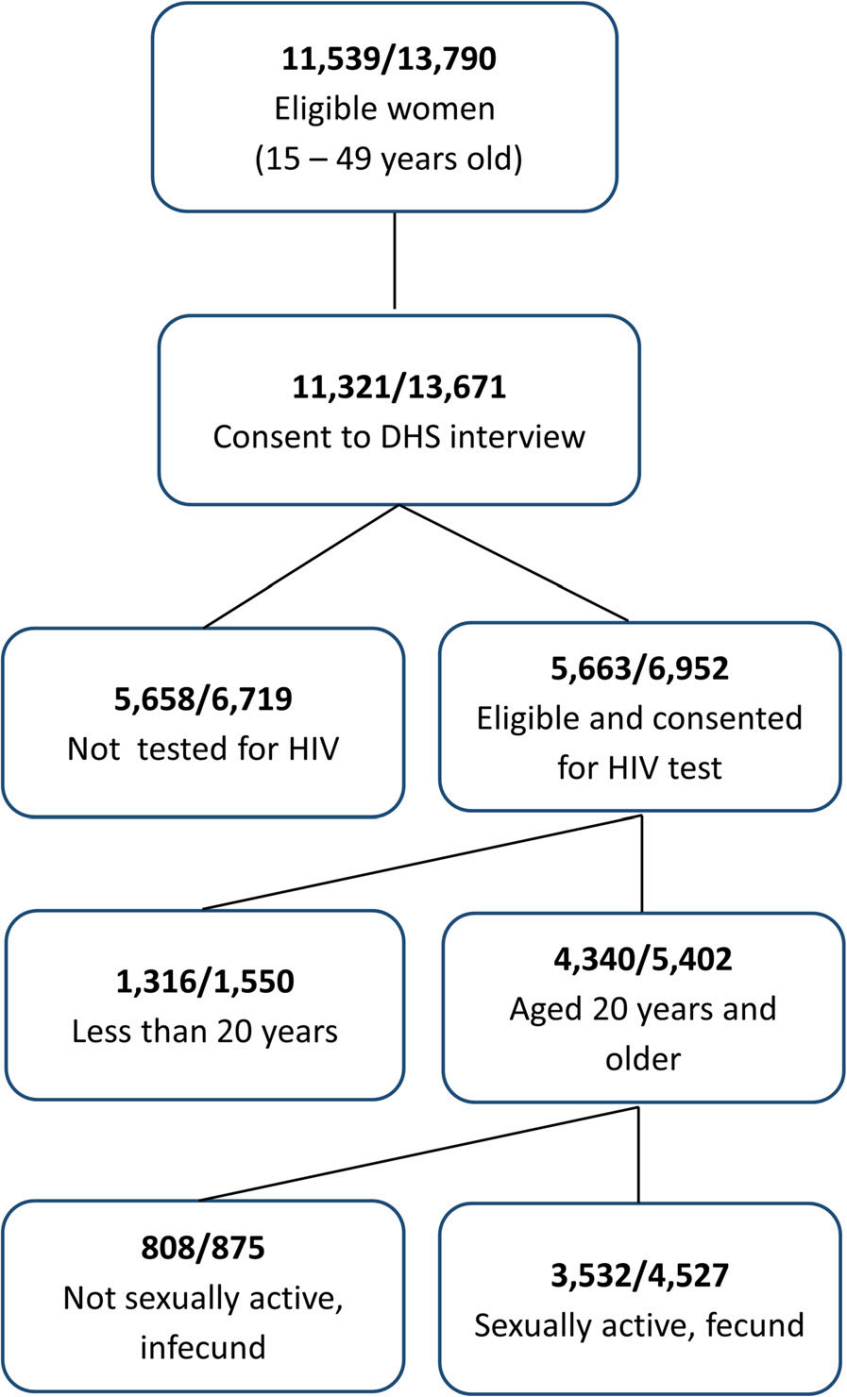
Flow chart of individuals included in the analyses of 2005 and 2010 (unweighted)

In the 2010 RDHS, HIV testing was offered to 6952 eligible women, 99% of whom consented. Questions about fertility, however, were only asked to women who self-reported ever having sex and not being infertile. Fertility in women under age 20 (41 births per 1000 women) is much lower and influenced by legal age of marriage (21 years in Rwanda), secondary school attendance, and other factors not applicable to woman age 20 to 24 (189 births per 1000 women) and older [11], thus this analysis excluded women less than 20 years of age. The final analysis included the 4527 women who consented to HIV testing, responded to the fertility questions, and were aged 20 or older (Fig. 2).

For a secondary analysis, the 2005 RDHS [12] was used to evaluate if the relationship between HIV and fertility changed between 2005 and 2010 which would provide evidence of unmeasured factors, such as ART scale up, changing between these two points in time. The 2005 RDHS was collected using the same methods as the 2010 RDHS. The 2005 RDHS included 11,539 women (15 to 49 years), and HIV testing was offered to 5663 women with 98.8% consent rate, resulting in 3532 women age 20 or older in the secondary analysis.

### Variables and analysis

**Fig. 3:**
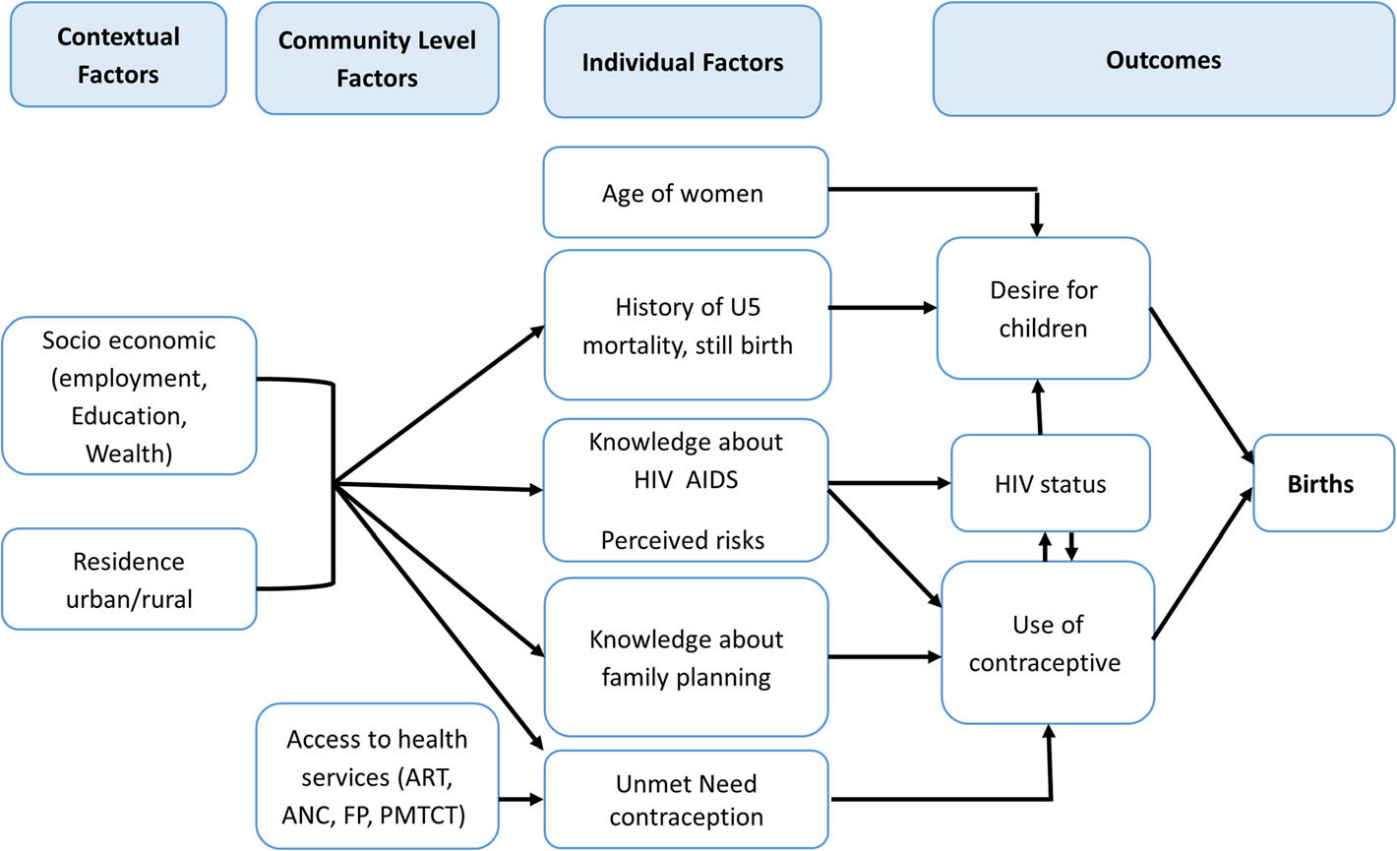
Conceptual framework

Two outcomes were explored. The first outcome of interest was fertility in the last 5 years defined as having given birth in the last 5 years or currently being pregnant. The second outcome of interest was a desire to have more children, defined as a woman reports that she would like to have a (another) child. A literature review and development of a conceptual framework informed key variables to include in this analysis (Fig. 3). Socio-demographic variables included in the analysis were wealth index, urban/rural residence, family size, polygamous marriage, woman’s educational level, and woman’s employment status. Clinical and behavioral data for the women included HIV status, knowledge of MTCT, number of lifetime sex partners, polygamous marriage, current use of contraception, and fertility more than 5 years ago. Variables identified in the conceptual framework that were not available in the dataset included type and timing of past sexually transmitted infection (STI), knowledge of HIV status, and use of antiretroviral therapy. Only those variables which changed the coefficient of the primary predictor, HIV status, in one or more age group by 10% or more were considered for the final model as a potential confounder.

HIV prevalence, fertility in the last 5 years, and desire to have more children were estimated with 95% confidence intervals for all covariates and chi-square tests were used to describe differences between groups. Multiple logistic regression models were used to assess the association between HIV and each of the two outcomes. Since fertility patterns and drivers change over the life course, the effect of HIV was modeled in three separate age groups: 20–29, 30–39 and 40–49 years by including an interaction term of HIV status and age group. All potential confounders that were not collinear (r<=|0.7|) were included in the final multivariable model, and records with any missing data were dropped. We assessed each covariate for confounding the primary effects, and included it in the final model if it changed the effect of HIV within any age group by 10% or more. Models were built for each outcome separately.

For the secondary analysis, we produced multivariable regression models for 2005 as a comparison and presented adjusted odds ratios (AOR). In these 2005 models, we controlled for the same confounders identified in the 2010 analysis to ensure comparability. All analyses were completed in Stata v12 using the *svy* commands to account for sample weights, clustering of households, and stratification by district (2010) or province (2005).

### Ethics

For both the 2005 and 2010 RDHS, informed consent was obtained from every study participant before the start of the interview, and a second consent was obtained before blood draw to test for HIV. The RDHS received ethical approval from the Rwandan National Ethics Committee and the US Centers for Disease Control and Prevention (CDC). We received permission from the MeasureDHS project to download and use these data for this analysis.

## Results

The overall prevalence of HIV in 2010 was 5.2% (95% CI: 4.6%, 6.0%) (Table 1). In the bivariate analysis, both fertility in the last 5 years and desire for more children were significantly associated with HIV (*p* < 0.001; Table 2). Significantly higher rates of HIV were observed among women who had at least a secondary education, were employed, had higher household wealth, knew about MTCT, or lived in urban areas. Women with an unmet need for contraception and women who had a larger number of births more than 5 years ago had a lower prevalence of HIV (Table 2).

**Table 1:** HIV prevalence in Rwanda by background characteristic, 2010

Characteristic	N (weighted)	HIV Prevalence	95% CI	*X* ^2^ *p*-value
Overall	4549	5.2	[4.6,6.0]	--
Fertility in the last 5 years				
No children	1270	8.6	[7.2,10.2]	<0.001
1–4 children, or pregnant	3279	3.9	[3.3,4.7]	
Desire more children				
No more, sterilized	2466	6.7	[5.7,7.8]	<0.001
Want another, undecided	2083	3.5	[2.7,4.5]	
Age group				
20–29	1903	4.2	[3.4,5.3]	0.037
30–39	1541	6.0	[4.9,7.3]	
40–49	1105	5.9	[4.7,7.5]	
Residence				
Urban	681	12.2	[9.9,14.9]	<0.001
Rural	3868	4.0	[3.4,4.7]	
Education				
Less than secondary	4036	4.8	[4.1,5.5]	<0.001
Secondary or higher	513	9.0	[6.9,11.7]	
Employment^a^				
Not working, farmer	3860	4.4	[3.7,5.1]	<0.001
Employed	681	10	[8.0,12.5]	
Wealth group				
Bottom 2 quintiles	1847	4.5	[3.6,5.7]	<0.001
Middle quintile	889	3.5	[2.4,5.0]	
Top 2 quintiles	1813	6.8	[5.7,8.2]	
Number of lifetime sex partners^a^				
1 partner	3247	3.0	[2.5,3.7]	<0.001
2–4 partners	1257	10.1	[8.4,12.1]	
5+ partners	42	30.1	[17.6,46.3]	
Polygamous marriage^a^				
No	3109	3.3	[2.8,4.0]	<0.001
Yes	267	5.9	[3.5,9.6]	
Not married	1158	10.1	[8.5,12.0]	
Fertility more than 5 years ago				
No birth	361	4.3	[2.5,7.2]	<0.001
1–3 births	1297	7.7	[6.3,9.5]	
4–6 births	1820	4.9	[4.0,5.9]	
7+ births	1071	3.1	[2.2,4.4]	
Age at first sex^a^				
< 21	2748	60.5	[58.9,62.0]	0.039
21–24	1352	29.8	[28.4,31.2]	
25+	444	9.8	[8.9,10.7]	
Ever had a terminated pregnancy^a^				
No	3725	5.3	[4.6,6.1]	0.858
Yes	822	5.1	[3.8,6.8]	
Ever had a death of child under 5				
No death	3164	4.6	[3.9,5.4]	0.008
Lost one or more children under 5	1385	6.7	[5.3,8.4]	
Current use of modern method of contraception				
No	2825	5.2	[4.4,6.2]	0.965
Yes	1724	5.3	[4.2,6.5]	
Has an unmet need for contraception^a^				
No	3792	5.7	[5.0,6.5]	0.002
Yes	754	3.0	[2.0,4.5]	
Knows of a modern contraceptive method^a^				
No	7	12.8	[1.7,56.2]	0.356
Yes	4541	5.2	[4.6,6.0]	
Knowledge of MTCT^a^				
No	326	2.5	[1.2,4.8]	0.019
Yes	4191	5.5	[4.8,6.3]	
Comprehensive knowledge of HIV/AIDS				
No	1957	4.2	[3.5,5.2]	0.003
Yes	2592	6.0	[5.1,7.0]	

^a^Categories may not sum to 100% due to missing data

**Table 2:** Distribution of fertility in the last 5 years and desire to have more children in Rwanda by background characteristic, 2010

		Fertility in the last 5 years			Desire more children		
Characteristic	n (weighted)	Percent	95% CI	*X* ^2^ *p*-value	Percent	95% CI	*X* ^2^ *p*-value
Overall	4549	72.1	[70.6,73.6]		45.8	[44.3,47.3]	
HIV status							
Negative	4311	73.1	[71.5,74.6]	<0.001	46.6	[45.1,48.2]	<0.001
Positive	238	54.3	[47.9,60.6]		30.5	[24.6,37.2]	
Age group							
20–29	1903	86.4	[84.4,88.2]	<0.001	76.3	[74.4,78.2]	<0.001
30–39	1541	76.7	[74.4,78.9]		33.3	[30.7,35.9]	
40–49	1105	40.9	[38.0,43.9]		10.6	[8.9,12.6]	
Residence							
Urban	681	63.2	[58.9,67.3]	<0.001	51.9	[47.8,56.1]	0.002
Rural	3868	73.6	[72.0,75.2]		44.7	[43.1,46.3]	
Education							
Less than secondary	4036	73.3	[71.8,74.8]	<0.001	44.5	[43.0,46.1]	<0.001
Secondary or higher	513	62.4	[57.6,66.9]		55.5	[51.0,59.9]	
Employment^a^							
Not working, farmer	3860	74.0	[72.4,75.5]	<0.001	44.0	[42.4,45.7]	<0.001
Employed	681	61.9	[57.9,65.7]		55.7	[51.6,59.7]	
Wealth group							
Bottom 2 quintiles	1847	76.8	[74.8,78.8]	<0.001	41.9	[39.5,44.4]	<0.001
Middle quintile	889	73.3	[69.9,76.5]		47.4	[44.1,50.7]	
Top 2 quintiles	1813	66.6	[64.2,69.0]		48.9	[46.4,51.4]	
Number of lifetime sex partners^a^							
1 partner	3247	74.8	[73.1,76.5]	<0.001	48.9	[47.2,50.7]	<0.001
2–4 partners	1257	66.2	[63.5,68.9]		37.7	[34.8,40.7]	
5+ partners	42	35.5	[22.3,51.3]		44.2	[29.1,60.6]	
Polygamous marriage^a^							
No	3109	81.3	[79.7,82.7]	<0.001	48.3	[46.5,50.1]	<0.001
Yes	267	72.7	[67.0,77.8]		29.8	[24.5,35.7]	
Not married	1158	47.4	[44.4,50.4]		43.0	[44.3,47.4]	
Fertility more than 5 years ago							
No birth	361	29.9	[24.9,35.4]	<0.001	96.0	[93.5,97.6]	<0.001
1–3 births	1297	71.1	[68.4,73.7]		71.4	[68.6,74.0]	
4–6 births	1820	76.7	[74.6,78.6]		37.4	[35.0,39.9]	
7+ births	1071	79.6	[76.9,82.1]		12.1	[10.4,14.2]	
Age at first sex^a^							
< 21	2748	58.9	[57.0,60.8]	<0.001	41.1	[39.2,43.0]	<0.001
21–24	1352	48.7	[46.0,51.5]		51.3	[48.5,54.0]	
25+	444	42.0	[37.4,46.8]		58.0	[53.2,62.6]	
Ever had a terminated pregnancy^a^				<0.001			<0.001
No	3725	73.6	[72.0,75.2]		48.4	[46.7,50.0]	
Yes	822	65.0	[61.6,68.3]		34.1	[30.8,37.5]	
Ever had a death of child under 5							
No death	3164	73.5	[71.7,75.2]	<0.001	53.9	[52.1,55.8]	<0.001
Lost one or more children under 5	1385	68.8	[66.2,71.2]		27.2	[24.8,29.7]	
Current use of modern method of contraception							
No	2825	65.3	[63.2,67.2]	<0.001	46.6	[44.6,48.6]	0.212
Yes	1724	83.3	[81.3,85.1]		44.5	[42.1,46.9]	
Has an unmet need for contraception^a^							
No	3792	67.7	[66.0,69.3]	<0.001	47.8	[46.1,49.4]	<0.001
Yes	754	94.1	[92.1,95.6]		35.8	[32.3,39.5]	
Knows of a modern contraceptive method^a^							
No	7	40.0	[10.3,79.4]	0.101	70.9	[30.2,93.2]	0.207
Yes	4541	72.1	[70.6,73.6]		45.7	[44.2,47.3]	
Knowledge of MTCT^a^							
No	326	65.7	[59.9,71.0]	0.011	43.4	[38.3,48.6]	0.374
Yes	4191	72.8	[71.2,74.4]		45.9	[44.3,47.5]	
Comprehensive knowledge of HIV/AIDS							
No	1957	72.9	[70.6,75.0]	0.350	45.2	[43.1,47.4]	0.537
Yes	2592	71.5	[69.5,73.4]		46.2	[44.1,48.3]	
Desire more children							
No more, sterilized	2466	67.4	[65.4,69.3]	<0.001			
Want another, undecided	2083	77.6	[75.4,79.7]				
Birth in the last 5 years, or pregnant							
No children	1270				36.7	[33.8,39.7]	<0.001
1–4 children or pregnant	3279				49.3	[47.5,51.1]	

^a^Categories may not sum to 100% due to missing data

Overall, seventy two percent (72.1%) of the women in 2010 (95% CI: 70.6%, 73.6%) had at least one birth in the last 5 years or were currently pregnant, and 45.8% (95% CI: 44.3%, 47.3%) desired to have more children (Table 2). In bivariate analysis, HIV seropositive women were less likely to have given birth to a child in the last 5 years or be pregnant (54.3%, 95% CI: 47.9%, 60.6%) and less likely to desire children in the future (30.5%, 95% CI: 24.6%, 37.2%) compared to HIV seronegative women (73.1%, 95% CI: 71.5%, 74.6%; and 46.6%, 95% CI: 45.1%, 48.2%, respectively). The factors significantly associated with recent fertility in the bivariate analysis were: age group, residence, education, employment, wealth group, number of lifetime sex partners, polygamous marriage, fertility more than 5 years prior to the survey, age at first sex, ever had a terminated pregnancy, every had a death of a child under 5, current use of a modern method of contraception, unmet need for contraception, knowledge of MTCT, and desire for more children (Table 2). The factors significantly associated with the desire for more children in the bivariate analysis were: age group, residence, education, employment, wealth group, number of lifetime sex partners, polygamous marriage, fertility more than 5 years prior to the survey, age at first sex, ever had a terminated pregnancy, ever had a death of a child under 5, has an unmet need for contraception, had a birth in the last 5 years or is currently pregnant.

After controlling for covariates and applying an interaction term between HIV and age group, fertility no longer differed statistically between HIV seropositive and HIV seronegative women (Table 3). The odds of having a recent birth or being pregnant was lower but non-significant in younger infected women (20–29, 30–39 years) compared to their uninfected counterparts. In regression analysis, HIV seropositive women were less likely to desire more children compared to uninfected women among 20 to 29 year olds (AOR = 0.31, 95% CI: 0.17, 0.58) and 30 to 39 years (AOR = 0.24, 95% CI: 0.14, 0.43). There were no differences in desire for more children among women age 40 to 49 (AOR = 1.57, 95% CI: 0.75, 3.27, Table 3).

**Table 3:** Effect of HIV status on fertility and desire of more children in Rwanda, 2010

		Birth in the last 5 years or pregnant^a^		Desire more children^b^	
		AOR	95% CI	AOR	95% CI
Age 20–29	HIV-	Ref		Ref	
	HIV+	0.83	[0.42, 1.65]	0.31	[0.17, 0.58]
Age 30–39	HIV-	Ref		Ref	
	HIV+	0.60	[0.35, 1.03]	0.24	[0.14, 0.43]
Age 40–49	HIV-	Ref		Ref	
	HIV+	1.13	[0.48, 2.67]	1.57	[0.75, 3.27]
n		4501		4466	

^a^Confounders controlled for: Woman’s employment, woman’s education, number of lifetime sex partners, polygamous marriage, household wealth, urban/rural residence, had a death of a child under 5, unmet need for contraception, desire more children

^b^Confounders controlled for: Woman’s employment, woman’s education, number of lifetime sex partners, polygamous marriage, household wealth, urban/rural residence, age at first sex, ever terminated a pregnancy, knowledge of MTCT, had a death of a child under 5, use of contraception, unmet need for contraception, birth in the last 5 years or pregnant

When the same models were run with the 2005 data, no associations were identified between HIV status and fertility in the last 5 years in any age group (Table 4). There were no associations between births in the last 5 years and HIV status (20–29 years: AOR = 0.43, 95% CI: 0.13, 1.51; 30–39 years: AOR = 1.33, 95% CI: 0.62, 2.84; and 40–49 years: AOR = 1.35, 95% CI: 0.63, 2.91). Similarly, we did not identify association between HIV status and desire for more children (20–29 years: AOR = 0.62, 95% CI: 0.30, 1.28; 30–39 years: AOR = 0.72, 95% CI: 0.44, 1.19; and 40–49 years: AOR = 0.90, 95% CI: 0.36, 2.26).

**Table 4:** Effect of HIV status on fertility and desire of more children in Rwanda, 2005

		Birth in the last 5 years or pregnant^a^		Desire more children^b^	
		AOR	95% CI	AOR	95% CI
Age 20–29	HIV-	Ref		Ref	
	HIV+	0.43	[0.13, 1.51]	0.62	[0.30, 1.28]
Age 30–39	HIV-	Ref		Ref	
	HIV+	1.33	[0.62, 2.84]	0.72	[0.44, 1.19]
Age 40–49	HIV-	Ref		Ref	
	HIV+	1.35	[0.63, 2.91]	0.90	[0.36, 2.26]
n		3524		3366	

^a^Confounders controlled for: Woman’s employment, woman’s education, number of lifetime sex partners, polygamous marriage, household wealth, urban/rural residence, had a death of a child under 5, unmet need for contraception, desire more children

^b^Confounders controlled for: Woman’s employment, woman’s education, number of lifetime sex partners, polygamous marriage, household wealth, urban/rural residence, age at first sex, ever terminated a pregnancy, knowledge of MTCT, had a death of a child under 5, use of contraception, unmet need for contraception, birth in the last 5 years or pregnant

## Discussion

The relationship between HIV status and women’s fertility is complex, both biologically and behaviorally. These secondary analyses have shown that, after controlling for other factors in the multiple logistic regression models, the differences in fertility for the last 5 years between HIV seropositive women as compared to seronegative women were not statistically significant. However, the desire for more children in the future is lower among HIV seropositive women aged 20 to 39 compared to HIV seronegative women in Rwanda in 2010.

### The relationship between HIV and fertility

The literature about the relationship between HIV infection and fertility is conflicting. The notion that HIV would lead to a stark decrease in fertility has been challenged by evidence that HIV infected women actually have more children than their HIV seronegative peers [13]. A study conducted in Kenya in 2010 showed an increase in fertility in HIV infected women due to an increased mortality in children which led HIV infected women to have more children [14]. However, the increase in fertility in HIV seropositive women may only be true for those women who are still in the early stage of their infection. There is increasing evidence suggesting that HIV infected women, particularly women with advanced HIV infections or AIDS, are less fertile than HIV uninfected women [15].

One possible explanation for no significant association between HIV and fertility in 2010 is the scale-up of ART. In Rwanda, it was estimated that 85% of HIV infected adults in need were on ART in 2010 (compared to 44% in 2005), and the median CD4 cell count at ART initiation among adult patients increased from 153 cells/mm^3^ in 2005 to 277 cells/mm^3^ in 2010 [Unpublished dissertation by Dr. Jean Pierre Nyemazi (2012) Outcomes of adult patients on Antiretroviral Therapy from 2004 to 2010 in National University of Rwanda, School of Public Health]. Thus expanded access to ART in Rwanda may explain the lack of significant differences in fertility that we observed in this analysis. Recent studies have reported an independent effect of ART on increasing pregnancy incidence over time in women on treatment compared with ART-naıve women [5, 6, 16]. In several settings where antiretroviral therapy has been scaled-up, pregnancy and births rates among HIV seropositive women have significantly increased [17]. The improved individual health through the availability of ART may result in improved sexual drive, influencing the behavior of HIV seropositive women [18]. Women on treatment may feel healthier, more optimistic about the PMTCT interventions, more positive about their own and children’s futures, and therefore engage in unprotected sexual activity and be more inclined to become pregnant [19].

Another possible explanation for the lack of a significant difference in fertility between HIV seropositive and HIV seronegative women in 2010 is the emphasis that Rwanda has put on promoting access and use of family planning services. Over the last 10 years, the country experienced a remarkable increase in modern contraceptive use. The contraceptive prevalence rate increased from 17 to 52% between 2005 and 2010 [11, 12]. Unmet need for family planning declined from 38 to 19%. The infant mortality rate has decreased from 105 deaths to 50 deaths per 1000 live births, and the total fertility rate dropped from 6.1 to 4.6 births [12, 20]. Thus in addition to increased fertility of HIV seropositive women, there may also be a greater drop in fertility among HIV seronegative women due to use of modern contraceptive methods.

A third potential explanation is that there is no relationship between HIV and fertility in Rwanda, which is supported by the results of the 2005 analysis. However, given the large improvements in survival with HIV, the lack of relationship in 2005 is likely influenced by survivor bias; women who were so sick they could not or chose not be become pregnant did not survive to be measured.

### The relationship between HIV and the desire for more children

HIV infected women between 20 and 39 years of age were significantly less likely to want more children in the future than those not infected with HIV in 2010. This was similarly shown in some studies [21, 22], but not all [23]. There are several hypotheses for this finding. In the context of high service coverage, women infected with HIV have monthly contact with the health system through pre-ART or ART care to obtain medication. Therefore, with integrated HIV care and treatment services with family planning services, it is highly conceivable that through regular contact with the health system, infected women have increased awareness about the benefits of spacing and small family sizes thus decreasing their desire for more children. More research is needed to explore the plausibility of this hypothesis.

A competing hypothesis is that the lower desire for children among HIV seropositive women may be born from stigma or fear. In other studies, reasons for HIV infected women not wanting another child included the need to provide for their living children; fear that their poor health may prevent them from taking care of an additional child; the fear of leaving the child orphaned; the fear that they may not have any family support to raise the child; the fact that they were not yet fully convinced about the efficacy of antiretroviral triple therapy in mitigating MTCT; and fear that the physical demands of pregnancy and childbirth could negatively impact their own health [18, 22, 24].

We did not observe a similar effect between HIV and fertility desire in the 2005 secondary analysis. However, this can reflect both survivor bias, since very few people were on HIV treatment at the time, and the very low prevalence of women who knew their HIV status at that time (11.6%) [12].

### Limitations

This study used data available through two recent RDHSs. With the cross-sectional nature of the data collection, it is impossible to ascertain the sequence of outcomes and exposures, specifically the length of HIV infection, whether or not a woman knew her infection status (either at the time of the survey or the time of pregnancy), and whether or not she was accessing HIV care. Our hypothesis that access to ART contributed to the non-significant difference in fertility rates between HIV infected and HIV uninfected women relies on the assumption that HIV infected women knew their status and accessed HIV care and treatment services. This is plausible in 2010 when an estimated 80% of eligible individuals in need were on ART, but tenuous in the full 5 years under study as these map to years of scale-up of the ART program. The amount that ART is contributing to non-differential fertility rates can be further explored by repeating this analysis with the 2015 RDHS underway at the time of this analysis, and the 2013 Rwanda AIDS indicator survey. The hypothesis that knowledge of HIV status decreases desire for more children (either through increased family planning knowledge or because of stigma/fear) hinges on the assumption that a woman knew her status at the time of the RDHS data collection. This is a plausible assumption given that in 2010, 77.2% of respondents had ever had an HIV test and 38.6% had a test in the last 12 months [11]. Another limitation resulting from use of secondary data was that not all variables contributing to the relationship between HIV and fertility were available. Specifically, data on past STIs and antiretroviral therapy use are important confounders that were unavailable for this analysis. We recommend that future iterations of the RDHS collect symptoms of common STIs to explore these hypotheses further, though current DHS policy prevents directly asking respondents to disclose HIV status by naming use of ART.

## Conclusion

Non-significant differences in fertility between infected and uninfected women suggest progress in addressing the health and social consequences of HIV. However, significant differences in desire for more children remain between these two groups. More research is needed to understand the factors influencing fertility desires among people living with HIV/AIDS and to test whether the availability of expanded HIV services including Option B+ introduced in 2012 in Rwanda has changed the fertility and fertility desires of HIV infected women. After the 2015 RDHS is released, it will be important to investigate changes in fertility and HIV between 2005, 2010, and 2015, as ART and PMTCT services expanded to include universal access to treatment in the general population and Option B+ for pregnant women.

## Abbreviations

ART: Antiretroviral therapy
CDC: Centers for Disease Control and Prevention
DHS: Demographic and Health Survey
HIV: Human immunodeficiency virus
PMTCT: Prevention of mother-to-child transmission
STI: Sexually transmitted infection
